# Acute Q Fever Patients Requiring Intensive Care Unit Support in Tropical Australia, 2015–2023

**DOI:** 10.3201/eid3102.240422

**Published:** 2025-02

**Authors:** Cody Price, Simon Smith, Jim Stewart, Josh Hanson

**Affiliations:** Cairns Hospital, Cairns, Queensland, Australia (C. Price, S. Smith, J. Stewart, J. Hanson); University of New South Wales, Sydney, New South Wales, Australia (J. Hanson)

**Keywords:** Q fever, *Coxiella burnetii*, critical care, intensive care unit, bacteria, zoonoses, tropical, Queensland, Australia

## Abstract

Acute Q fever is classically described as a mild illness. We report 9 patients with acute Q fever in Queensland, Australia, who required intensive care unit support to survive. Clinicians should consider an acute Q fever diagnosis and its empirical treatment in critically ill persons in the appropriate clinical context.

Q fever, a zoonotic bacterial disease caused by *Coxiella burnetii*, has a global distribution (*1*). Most acute *C. burnetii* infections are asymptomatic or manifest as a self-limiting, nonspecific febrile illness. Respiratory and gastrointestinal symptoms can also occur, which might necessitate hospitalization, but severe, life-threatening acute disease is reported rarely (*1*,*2*). In 1 large study, only 3 (0.2%) of 1,806 patients with acute Q fever died; 2 of those deaths were because of underlying malignancy (*2*). We report the cases of 9 patients from Queensland in tropical Australia with laboratory-confirmed acute Q fever who required intensive care unit (ICU) support to survive their infection. The Far North Queensland Human Research Ethics Committee provided ethics approval for the study (approval no. EX/2023/QCH/95302–1707QA). Because the retrospective data were deidentified, the committee waived the requirement for informed consent.

## The Study

Q fever is a notifiable disease in Australia. We used Queensland’s notifiable conditions database and electronic laboratory reporting system to identify all cases of acute Q fever in the Far North Queensland (FNQ) region during January 1, 2015–December 31, 2023. We only included cases meeting definitive laboratory criteria for acute Q fever: positive PCR or seroconversion or >4-fold increase in antibody titer to phase II antigen in paired serum samples (*3*). We recorded the patients’ demographic data and clinical, laboratory, and radiologic findings. We used the Charlson Comorbidity Index to quantify comorbidity; severe comorbidity was defined as a score of >5 (*4*). For patients with available data, we recorded the Queensland Adult Deterioration Detection System score, a vital signs–based early warning score, which was calculated when patients were first seen (*5*). We performed statistical analysis using Stata 18.0 (Stata, https://www.stata.com) and compared groups by using logistic regression and the Wilcoxon rank-sum, χ^2^, and Fisher exact tests, as appropriate. 

A total of 223 cases of Q fever in the FNQ region were reported to the notifiable diseases database during the study period; 127/223 (57%) patients sought care at a hospital in the region, 105/127 (83%) were admitted as inpatients, and 9/105 (9%) were admitted to the ICU (Table 1). Eight (89%) of the 9 patients requiring ICU admission lived in a rural location. None of the 9 patients had classical occupational exposure history, and none were known to be vaccinated against Q fever; 8/9 (89%) reported close contact with animals. Eight (89%) of 9 were >50 years of age, but only 1 (11%) had severe comorbidity. Only 1/9 (11%) was first seen within 7 days of symptom onset, but 7/9 (78%) had been prescribed antimicrobial drug therapy with activity against *C. burnetii* for >24 hours before their ICU admission. One otherwise healthy 55-year-old woman had received doxycycline for 4 days before her ICU admission (Appendix Table 1).

**Table 1:** Demographic and clinical characteristics of patients in study of acute Q fever infections requiring intensive care unit support in tropical Australia, 2015–2023*

Variable	Total no. patients with data†	All patients	Required ICU admission, n = 9	No ICU admission required, n = 118	p value
Median age, y (IQR)	127	54 (44–65)	60 (53–69)	54 (43–64)	0.14
Patient sex					
M	127	96 (76)	6 (67)	90 (76)	0.69
F	127	31 (24)	3 (33)	28 (24)	0.69
First Nations Australian‡	127	12 (9)	3 (33)	9 (8)	0.04
Rural residence	127	106 (84)	8 (89)	99 (84)	1.0
Vaccinated against *Coxiella burnetii*	58	0	0	0	NA
Immunocompromised	127	5 (4)	0	5 (4)	1.0
Pregnant female patients	31	0	0	0	NA
Median initial symptom duration, d (IQR)	124	5 (3–7)	7 (7–8)	5 (3–7)	0.055
Supplemental oxygen required§	57	6 (11)	2 (22)	4/48 (8)	0.24
Median systolic blood pressure,§ mm Hg (IQR)	57	130 (120–141)	122 (100–136)	131 (123–141)	0.051
Median heart rate,§ beats/min (IQR)	57	94 (77–104)	100 (90–115)	93 (74–104)	0.14
Median body temperature,§ °C (IQR)	57	37.4 (36.8–38.4)	37.2 (36.8–37.8)	37.9 (36.7–38.6)	0.71
Impaired consciousness§	56	0	0	0/47	NA
Early warning score (Q-ADDS)¶	57	2 (1–4)	4 (2–8)	2 (1–3)	0.01
Hepatitis#	127	79 (62)	2 (22)	77 (65)	0.03
Both hepatitis and pneumonia#**	127	33 (26)	6 (67)	28 (24)	0.01
Elevated cardiac biomarkers	21	6 (29)	2/6 (33)	4/15 (27)	1.0
Abnormal chest radiograph§	104	27 (26)	5 (56)	22/95 (23)	0.049
Died	127	0	0	0	NA

*Values are no. (%) except as indicated. p values compare the number of patients requiring ICU admission with the number of patients not requiring ICU admission for each variable. IQR, interquartile range; NA, not applicable; Q-ADDS, Queensland Adult Deterioration Detection System. †Retrospective nature of this study precluded collection of complete data for each variable, especially for vital signs reported when the patient was first seen. ‡All patients seeking care in the Australia public health system are asked whether they identify as a First Nations Australian (Aboriginal or Torres Strait Islander). §When patients were first seen. ¶Early warning score using vital signs, calculated on initial review. #Hepatitis defined as a peak of elevated transaminase enzymes >2 times the upper limit of reference range during the patient’s illness. **Pneumonia defined as dyspnea, cough, or hemoptysis or radiographic abnormalities.

The small sample size and retrospective nature of the study precluded detailed statistical analysis; however, patients requiring ICU care were more likely to have multiorgan involvement (odds ratio [OR] 5.42 [95% CI 1.21–24.31]; p = 0.03), an abnormal chest radiograph (OR 4.15 [95% CI 1.02–16.80]; p = 0.046), and an elevated early warning score (OR 5.42 [95% CI 1.21–24.31]; p = 0.03) when they were first seen (Tables 1, 2). Testing for serum antiphospholipid antibodies was performed for only 1 ICU patient (case no. 5) (Appendix); the result was positive. Three patients not requiring ICU care had serum samples tested for antiphospholipid antibodies; 1 result was negative, and 2 results were borderline positive.

**Table 2:** Laboratory findings when patients were first seen in study of acute Q fever infections requiring intensive care unit support in tropical Australia, 2015–2023*

Variable	No. patients with data†	All patients, n = 127	Required ICU admission, n = 9	ICU admission not required, n = 118	p value
Hemoglobin, g/dL	127	144 (133–155)	157 (133–163)	144 (133–154)	0.21
Leukocyte count, × 10^9^ cells/L	127	5.4 (4.0–6.9)	6.1 (5.4–7.7)	5.3 (4.0–6.8)	0.08
Neutrophil count, × 10^9^ cells/L	127	3.9 (2.7–5.2)	4.8 (4.1–5.9)	3.7 (2.5–5.0)	0.04
Platelet count, × 10^9^/L	137	120 (83–165)	93 (46–148)	121 (85–166)	0.14
Sodium, mmol/L	127	132 (130–135)	125 (125–133)	132 (130–135)	0.02
Potassium, mmol/L	127	3.9 (3.6–4.1)	4.0 (3.6–4.1)	3.9 (3.6–4.2)	0.74
Creatinine, μmol/L	127	84 (71–98)	86 (75–103)	84 (71–97)	0.51
Alanine aminotransferase, IU/L	127	103 (64–175)	125 (53–162)	102 (64–177)	0.97
Aspartate aminotransferase, IU/L	127	102 (71–167)	164 (56–251)	101 (72–165)	0.31
Total bilirubin, μmol/L	127	16 (10–22)	20 (16–58)	16 (10–22)	0.07
Serum alkaline phosphatase, IU/L	127	91 (70–149)	124 (93–172)	88 (68–148)	0.06
Gamma-glutamyl transferase, IU/L	127	78 (36–164)	107 (48–272)	75 (36–152)	0.33
Lactate dehydrogenase, IU/L	124	456 (364–604)	394 (356–673)	456 (362–593)	0.94
Prothrombin time, s	47	14 (12–15)	15 (13–17)	13 (12–15)	0.17
Activated partial thromboplastin time, s	46	32 (29–36)	35 (29–40)	31 (29–36)	0.30
Fibrinogen, g/L	46	4.1 (3.2–4.8)	3.6 (2.6–4.6)	4.2 (3.4–4.8)	0.13
Ferritin, μg/L	19	1,160 (835–2,065)	5,340 (2,260–8,420)	1,035 (752–1,678)	0.07
C-reactive protein, mg/L	115	133 (84–180)	158 (61–236)	132 (85–179)	0.64

*Values are median (interquartile range) except as indicated. p values compare the number of patients requiring ICU admission with the number of patients not requiring ICU admission for each variable. IU, international units. †The retrospective nature of the study precluded the collection of complete data for all cases.

The actual diagnosis of Q fever in the 9 patients requiring ICU admission was often delayed or even retrospective. Initial serologic results suggested acute Q fever in only 3/9 (33%) patients; those results were negative for 5/9 (55%) and suggested previous *C. burnetii* infection in 1/9 (11%) (Appendix Table 2). Serum PCR was positive in every case that was tested with that method; however, access to those PCR results was often delayed because testing was performed by the statewide reference laboratory, which was 1,390 km away. Indeed, 2 ICU patients were discharged from the hospital before their Q fever diagnosis was confirmed, and both patients received less than the recommended 14 days of antimicrobial drug therapy (7 and 10 days) (*6*).

The 9 patients were in the ICU for a median of 3 (interquartile range 2–5) days; 6/9 (67%) required vasopressor support for hypotension and 1/9 (11%) required mechanical ventilation (Figure), whereas 2/9 (22%) needed no organ support but required monitoring of multiorgan dysfunction (Appendix Table 1). No patient admitted to ICU required renal replacement therapy. Indeed, for a critically ill population, the patients’ renal function was remarkably preserved; the highest recorded serum creatinine in any of the 9 patients during their hospitalization was 123 µmol/L (Appendix Table 3).

**Figure:**
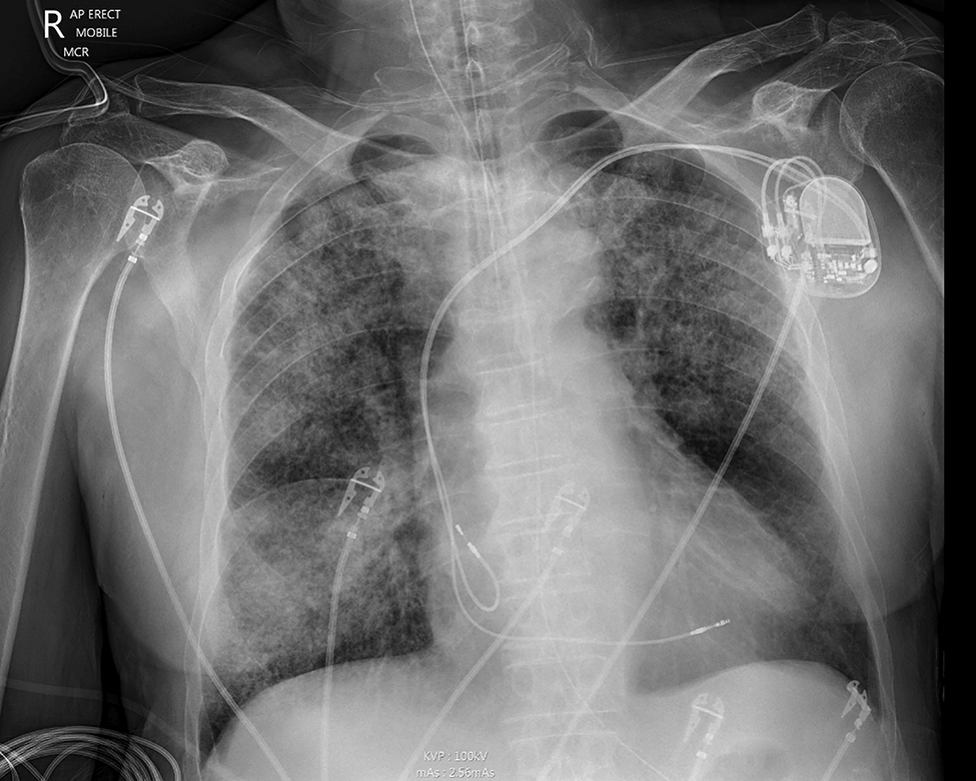
Chest radiograph of a patient (patient 7) showing right upper lobe opacification in study of acute Q fever infections in the intensive care unit in tropical Australia, 2015–2023. The patient required mechanical ventilation for 72 hours.

Patients requiring ICU admission spent a median of 11 (interquartile range 8–18) days in the hospital. All 9 ICU patients survived to hospital discharge, and none have subsequently received a diagnosis of chronic Q fever, although follow-up serologic testing has been performed for only 4/9 (44%) (Appendix). One patient required the insertion of a permanent pacemaker for atrioventricular block 8 months after his hospital admission, although this need was considered to be unrelated to his *C. burnetii* infection.

## Conclusions

Acute Q fever is classically thought to be a mild illness; however, 9/223 (4%) patients with a confirmed acute infection in the FNQ region of Australia required ICU care to survive their infection. Severe disease in those 9 patients might be explained by a delay in seeking medical care and in appropriate antimicrobial drug therapy; only 1 patient sought care within 7 days of symptom onset, and only 4 received antimicrobial drugs with activity against *C. burnetii* when they were first seen. The delay in effective therapy was partly explained by a lack of timely access to PCR results, which might have expedited initiation of targeted antimicrobial drug therapy and prevented some of the patients’ subsequent deteriorations. 

Acute Q fever can be life-threatening. Its complications include severe pneumonia, hepatitis, meningoencephalitis, and myocarditis (*1*); however, hypotension is rarely reported. It is, therefore, notable that 6 patients in this series required vasopressor support. We hypothesize that this hypotension was distributive and caused by sepsis because it responded relatively promptly to fluid resuscitation and antimicrobial drug therapy; vasopressor support was usually required for <72 hours (*7*). Severe disease and hypotension have not been a feature of large case series in Australia, although the clinical descriptions in those studies were frequently not detailed (*8*–*10*).

There is growing recognition of marked geographic variation in the clinical phenotype of acute Q fever, which might be explained by variation in lipopolysaccharide expression in different *C. burnetii* strains (*1*,*11*). Strain variation might, at least partly, explain the findings in our cohort. The presence of antiphospholipid antibodies during acute Q fever has also been associated with a complicated disease course (*2*) and were identified in the only patient admitted to ICU who had serum tested for antiphospholipid antibodies; this testing will now be performed routinely at our hospital. Expanded use of PCR to test for *C. burnetii* during the study period (available since 2016) might also have contributed to greater recognition of the severe clinical phenotype described in this cohort (Appendix Figures 1, 2).

In conclusion, acute Q fever can cause life-threatening disease in otherwise healthy persons, and the clinical phenotype can evolve even after effective antimicrobial drug therapy begins. PCR is a far more sensitive diagnostic test than serology during early *C. burnetii* infection, and a positive result enables prompt, potentially lifesaving therapy and enhanced follow-up to identify chronic disease.

Appendix Additional information for acute Q fever requiring intensive care unit support in tropical Australia, 2015–2023.
