# Supplementary material for: Acute Q Fever Patients Requiring Intensive Care Unit Support in Tropical Australia, 2015–2023
Source: Emerg Infect Dis. 2025 Feb;31(2):332–5. doi: 10.3201/eid3102.240422 (PMC11845150; doi:10.3201/eid3102.240422)
Supplement: Appendix — Additional information for acute Q fever requiring intensive care unit support in tropical Australia, 2015–2023. [file 24-0422-Techapp-s1.pdf]

Article DOI: https://doi.org/10.3201/eid3102.240422

EID cannot ensure accessibility for supplementary materials supplied by authors. Readers who have difficulty accessing supplementary content should contact the authors for assistance.

Acute Q Fever Requiring Intensive Care Unit Support in Tropical Australia, 2015–2023

Appendix

Case Vignettes

Case 1

A 73-year-old man attended hospital because of a 5-day history of fever, myalgia, headache, and vomiting on a background of localized prostate cancer. A chest radiograph demonstrated a right basal infiltrate, and he was treated with ceftriaxone and doxycycline as empirical cover for pneumonia and leptospirosis (because of his rural residence and deranged liver function tests). Despite this, on the second day of his admission, hypotension developed with a systolic blood pressure as low as 65mmHg, which was unresponsive to fluid resuscitation. Piperacillin/tazobactam was substituted for the ceftriaxone, noradrenaline treatment began, and he was admitted to the intensive care unit (ICU), where he stayed for 2 days receiving vasopressor support and high flow oxygen. His initial Q fever serologic test was negative, and PCR was not performed; however, he continued to improve on antimicrobial drug therapy and was discharged after 8 days in the hospital. He completed a 10-day course of doxycycline. Convalescent serology 2 weeks after seeking care demonstrated seroconversion (and negative tests for leptospirosis and rickettsial diseases) supporting the diagnosis of acute Q fever. Testing for chronic Q fever was not performed, but he had no clinical manifestations that would have been consistent with chronic infection in the following 4 years while under the care of oncologists for his prostate cancer, which, ultimately, caused his death.

Case 2

A 55-year-old, otherwise healthy woman attended hospital because of a 9-day history of fevers, myalgias, headache, abdominal pain, and rash after a recent tick bite. She was

prescribed doxycycline for a presumed rickettsial infection. Despite this, bilateral pneumonia and hepatitis with jaundice developed. Elevated troponin I and brain natriuretic peptide suggested myocardial injury, and she was admitted to the ICU for broad spectrum antimicrobial drug treatment (piperacillin/tazobactam was added to her doxycycline), oxygen via nasal prongs, and close observation. She spent 2 days in ICU but required no additional supportive care and was discharged to the general ward 2 days later. A transthoracic echocardiogram revealed no abnormality. A diagnostic rise in antibodies to the Q fever phase II antigen was demonstrated on paired serum samples collected 10 days apart, and a 14-day course of doxycycline was completed. She was discharged home after a total of 18 days in the hospital. Testing for chronic Q fever was not performed, but she remains well in the community and has not been hospitalized in the subsequent 7 years.

Case 3

A 60-year-old woman attended hospital because of a 2-week history of vomiting and diarrhea on a background of untreated Graves' hyperthyroidism and prior hazardous alcohol use. She was febrile, ataxic, hypokalemic, thrombocytopenic, coagulopathic and had transaminitis on liver function tests and right middle lobe consolidation on chest radiograph. Although she was treated with piperacillin/tazobactam, hypotension and tachypnoea developed, and she was admitted to ICU for fluid management, vasopressor support, and high flow oxygen via nasal prongs; doxycycline was added to her antimicrobial drug regimen. Fluid resuscitation unmasked anemia, and she received 2 units of packed erythrocytes and 2 units of platelets. She spent 48 hours in ICU and was discharged to the ward; her antimicrobial drug regimen was rationalized to doxycycline monotherapy for presumed community-acquired pneumonia, which was thought to have caused decompensation of undiagnosed chronic liver disease. A transthoracic echocardiogram revealed a mildly thickened mitral valve with mild regurgitation but no other abnormality. She was discharged home 4 days later having completed 7 days of doxycycline. Although Q fever PCR was performed on admission and was positive, this result was not available on discharge. Testing for chronic Q fever was not performed, but she remains well in the community and has not been hospitalized in the subsequent 6 years.

Case 4

A 50-year-old farm worker attended hospital because of a 1-week history of fever and jaundice. He was found to have new atrial fibrillation with thrombocytopenia, hepatitis, and hyponatremia. He was treated with ceftriaxone and doxycycline for a presumed zoonotic

infection; leptospirosis was thought most likely. Confusion, dyspnea, and an oxygen requirement developed, and he was admitted to ICU for high-flow oxygen and close monitoring. His antimicrobial drug regimen was escalated to meropenem and azithromycin. He spent 5 days in ICU where he slowly improved without any additional supportive care. Troponin I was tested 3 times during his ICU stay and was normal on each occasion. Q fever PCR on day 4 of his admission was reported as positive 5 days later, and he completed a 14-day course of azithromycin/doxycycline. Testing for chronic Q fever was not performed. He was discharged on metoprolol for rate control of his atrial fibrillation but was only intermittently adherent to this medication; he came back to the hospital 3 months later in atrial fibrillation with a rapid ventricular response and global hypokinesis of his left ventricle (estimated ejection fraction of 40%). After cardioversion and 3 months of bisoprolol, his left ventricular function returned to normal (61%), but a complete heart block developed 8 months after his Q fever hospitalization, which necessitated permanent pacemaker insertion. A transthoracic echocardiogram and cardiac magnetic resonance imaging before pacemaker insertion did not demonstrate any abnormality. The attending clinicians felt that his complete heart block was unrelated to *C. burnetii* infection.

Case 5

A 56-year-old woman attended hospital because of a 2-day history of fever and abdominal pain. She had a background of chronic obstructive pulmonary disease and poorly controlled type 2 diabetes mellitus. She was discharged from the emergency department (without antimicrobial drug therapy) for primary care follow-up after medical and surgical review and a reassuring computed tomography scan of her abdomen and pelvis. She came back to the hospital 9 days later with ongoing symptoms, persisting abnormal liver functions tests, and new-onset thrombocytopenia and coagulopathy. She had hypotension with a systolic blood pressure as low as 70 mmHg, which was unresponsive to fluid resuscitation and required vasopressor support with noradrenaline. She received empirical meropenem and doxycycline. Q fever serology had been requested on admission but was still pending when a Q fever PCR sample was collected on day 4 of her admission; her PCR test returned a positive result 3 days later. A transthoracic echocardiogram revealed a mildly thickened mitral valve with mild regurgitation but no other abnormality. She was discharged from ICU on day 5 of her admission and was discharged home 13 days after initial review, completing a 14-day course of doxycycline. Testing for chronic Q fever during follow-up was negative.

Case 6

A 65-year-old man attended hospital with a 10-day history of fever, cough, and malaise on a background of cirrhosis secondary to hazardous alcohol use and asthma. He had attended the emergency department 2 days before but had been discharged without specific therapy with a presumed viral illness. He returned to the hospital because he had ongoing symptoms with persistent fevers. Leptospirosis and melioidosis were thought the most likely possible diagnoses, and he was treated with empirical meropenem. He had 3 emergency calls on the ward for hypotension, tachycardia, and tachypnoea and he was admitted into ICU on the second day of his admission for noradrenaline support and noninvasive ventilation. Vancomycin and doxycycline were added to his antimicrobial drug regimen. He required a 9-day ICU admission for vasopressor support. Q fever PCR (sample collected 5 days after his initial symptoms) returned a positive result. He completed a 14-day course of doxycycline, and a transthoracic echocardiogram revealed no abnormality. His 7-week hospital admission was complicated by considerable deconditioning requiring in-hospital rehabilitation, but he had no long-term complications from his Q fever infection. Follow-up testing for chronic Q fever was negative.

Case 7

A 70-year-old man attended hospital with a 2-week history of intermittent chest pain and sweats on a background of hypertension and cerebrovascular disease. His initial chest radiograph demonstrated right upper lobe opacification, but despite meropenem and doxycycline, his respiratory status deteriorated rapidly, bilateral infiltrates developed, and he required mechanical ventilation for type 1 respiratory failure. Q fever PCR on the day of admission returned a positive result 7 days later, and his antimicrobial drug therapy was rationalized to complete a 14-day course of doxycycline. He required a total of 10 days in ICU, including mechanical ventilation for 72 hours, which was stepped down to high flow oxygen via nasal prongs. Nine days after discharge from ICU, a new oxygen requirement developed, and an electrocardiogram and echocardiogram were consistent with a Takotsubo cardiomyopathy with a left ventricular ejection fraction of 36%. He required a 5-week hospitalization to address his dyspnea and deconditioning; however, he made a complete recovery and a repeat echocardiogram 3 months after discharge showed complete resolution of the Takotsubo changes. Testing for chronic Q fever was not performed, but he had no symptoms of chronic Q fever in the respiratory clinic 18 months after his acute Q fever diagnosis.

Case 8

A 44-year-old man attended hospital because of a 1-week history of fever, cough, and headache on a background of hazardous alcohol use. He was hypotensive, having a systolic blood pressure as low as 80mmHg, which was unresponsive to fluid resuscitation and required vasopressor support with noradrenaline in the emergency department. He was admitted to the ICU 8 hours after seeking care. He was pancytopenic and hyponatremic, and liver function tests were deranged in a mixed pattern. He was treated presumptively for a tropical infection/Q fever with ceftriaxone and doxycycline. He was discharged from ICU after 24 hours, having required noradrenaline support for his blood pressure but no other organ support. A Q fever PCR sample had been collected by his general practitioner 24 hours before seeking hospital care and returned a positive result on day 5 of his admission. He was discharged home 6 days after seeking care and completed a 14-day course of doxycycline. He had no long-term complications from his Q fever infection. An echocardiogram was not performed. Testing for chronic Q fever during follow-up was negative.

Case 9

A 62-year-old man attended hospital because of a week of headache and fever on a background of hazardous alcohol use. He was febrile to 39.4°C and hypotensive with a systolic blood pressure as low as 79mmHg, which was unresponsive to fluid resuscitation. He required vasopressor support with noradrenaline in the emergency department and was admitted to ICU 8 hours after seeking initial care. His lumbar puncture revealed no abnormality. His liver function tests were deranged in a mixed pattern, and he was hyponatremic and thrombocytopenic. He initially received empirical ceftriaxone and azithromycin, which was adjusted to ceftriaxone and doxycycline after 24 hours. Q fever PCR on the day of his admission returned a positive result 5 days later. He was discharged from ICU after a 48 hour stay, having required intermittent noradrenaline support for his blood pressure but no other supportive care. He was discharged home after a 6-day hospitalization and completed a 14-day course of doxycycline. A transthoracic echocardiogram revealed no abnormality. He had no long-term complications from his Q fever infection. Testing for chronic Q fever during follow-up was negative.

References

1. Zhang GQ, Hotta A, Mizutani M, Ho T, Yamaguchi T, Fukushi H, et al. Direct identification of *Coxiella burnettii* plasmids in human sera by nested PCR. J Clin Microbiol. 1998;36:2210–3. PubMed https://doi.org/10.1128/JCM.36.8.2210-2213.1998

Appendix Table 1. Selected characteristics of the 9 patients with laboratory-confirmed acute Q fever who required ICU admission*

Case no.	Age, y/sex	Charlson Comorbidity Index	Comorbidities	Occupational or recreational exposure	Predominant clinical syndrome	Time from symptom onset to initial care, d	Time from initial care to effective antibiotics, d†	Time from initial care to ICU admission, d	Reason for ICU admission	ICU stay, d	Duration of hospitalization	Outcome
1	73/M	5	Localized prostate cancer, AF, obesity.	Rural residence, contact with kangaroos	Pneumonia. hepatitis	7	5	7	Hypotension (SBP 65) requiring vasopressor support	3	8 d	Survived; no complications
2	55/F	1	Crohn’s disease in remission	Lived on rural property multiple tick bites, contact with kangaroos	Pneumonia, hepatitis	9	0	4	Multiorgan dysfunction (pneumonia, jaundice, elevated troponin), for monitoring	2	18 d	Survived; no complications
3	60/F	2	Prior hazardous alcohol use	Rural residence	Pneumonia, hepatitis	7	5	5	Multiorgan dysfunction (confusion, pneumonia, hypotension [SBP 79], hepatitis, coagulopathy, profound hypokalemia); for vasopressor support and monitoring	2	10 d	Survived; no complications
4	50/M	1	Hypertension, hazardous alcohol use	Rural residence, worked at banana factory, contact with kangaroos	Pneumonia, hepatitis	7	0	1	Multiorgan dysfunction (new AF, hypoxia, jaundice, coagulopathy); for monitoring.	5	11 d	Survived; required PPM for AV block 8 mo after ICU discharge‡
5	56/F	3	Type 2 diabetes, COPD	Rural residence, kittens born in patients home 2 weeks before	Hepatitis	7	10	11	Hypotension (SBP 70) requiring vasopressor support	4	13 d	Survived; no complications
6	65/M	3	Asthma, MASLD	Lived on rural property with cattle,	Pneumonia, hepatitis	2	2	4	Hypotension (SBP 83) requiring	9	7 wk	Survived; no complications

Case no.	Age, y/sex	Charlson Comorbidity Index	Comorbidities	Occupational or recreational exposure	Predominant clinical syndrome	Time from symptom onset to initial care, d	Time from initial care to effective antibiotics, d†	Time from initial care to ICU admission, d	Reason for ICU admission	ICU stay, d	Duration of hospitalization	Outcome
				wallabies, and wild pigs					vasopressor support			
7	71/M	4	Hypertension, previous TIA, hazardous alcohol use	Lived on rural property with wallabies	Pneumonia, hepatitis	14	3	2	Respiratory failure requiring mechanical ventilation	10	6 wk	Survived; Takotsubo cardiomyopathy which resolved subsequently.
8	44/M	0	Nil	Lived on rural property. hand fed paddymelon	Pneumonia, hepatitis	7	0	1	Hypotension (SBP 80) requiring vasopressor support	2	6 d	Survived; no complications
9	62/M	3	Hazardous alcohol use	Lived on farm with many animals, rat infestation in home	Hepatitis	7	0	1	Hypotension (SBP 79) requiring vasopressor support	2	7 d	Survived; no complications

*Vasopressor support was with noradrenaline in all cases. AF, atrial fibrillation; AV, atrioventricular; COPD, chronic obstructive pulmonary disease; ICU, intensive care unit; MASLD, metabolic dysfunction-associated steatotic liver disease; PPM, permanent pacemaker; SBP, systolic blood pressure; TIA, transient ischemic attack.
†Antimicrobial drugs with activity against *Coxiella burnetii*.
‡Thought to be unrelated to his acute Q fever.

Appendix Table 2. *Coxiella burnetii*–specific diagnostic tests*

Case no.	Symptom duration before care, d	Initial PCR†	Time from initial care to PCR, d	Initial phase II antibody serology‡	Time from initial care to serologic test, d	Convalescent phase II antibody serology‡	Time between serologic tests, d§	Chronic Q fever testing‡
1	5	Negative	27	IgM and IgG negative by EIA	6	IgM and IgG reactive by EIA; IgG titer <1:10 by IFA	11	Not performed
2	10	Negative	24	IgM negative, IgG positive by EIA; IgG titer = 1:20 by IFA	0	IgM and IgG positive by EIA; IgG titer = 1:160 by IFA	10	Not performed
3	7	Positive	4	IgG and IgM titers ≥1:1,280 by IFA	4	Not performed	NA	Not performed
4	7	Positive	5	IgM and IgG negative by EIA	0	IgM and IgG positive by EIA; IgG titer ≥ 1:1,280 by IFA	8	Not performed
5	2	Positive	12	IgM positive, IgG negative by EIA	9	IgM positive, IgG equivocal by EIA; IgG titer ≥ 1:1,280 by IFA	2	Negative
6	10	Positive	5	IgM and IgG negative by EIA	2	IgM and IgG reactive by EIA; IgG titer = 1:320 by IFA	14	Negative¶

Case no.	Symptom duration before care, d	Initial PCR†	Time from initial care to PCR, d	Initial phase II antibody serology‡	Time from initial care to serologic test, d	Convalescent phase II antibody serology‡	Time between serologic tests, d§	Chronic Q fever testing‡
7	14	Positive	2	IgM positive, IgG equivocal by EIA; IgG titer ≥1:1,280 by IFA#	2	IgM and IgG reactive by EIA; IgG titer ≥ 1:1,280 by IFA	10	Not performed
8	7	Positive	−1**	IgM and IgG negative by EIA	0	Not performed	NA	Negative
9	7	Positive	0	IgM and IgG negative by EIA	0	IgM and IgG reactive; CFT titer = 1:256	22	Negative

*CFT, complement fixation test; EIA, enzyme immunoassay; IFA, immunofluorescence assay; NA, not applicable.
†PCR of patient samples performed at Cairns Hospital, Cairns, Queensland, Australia. During 2015–2022, a validated in-house assay was used with nested PCR primers designed to target the *com1* gene encoding a 27-kDa outer membrane protein (*1*). In 2023, a validated in-house real-time PCR assay was used to amplify the *IS111a* transposase gene.
‡Serologic testing of patient samples at Cairns Hospital. Initial serologic testing for Q fever occurs with a commercial EIA for IgG (EuroImmun, https://www.euroimmun.com) and IgM (NovaLisa; Gold Standard Diagnostics, https://clinical.goldstandarddiagnostics.com), performed according to the manufacturer's instructions. IgG-positive/equivocal or IgM-positive specimens undergo additional testing via IFA by using commercial antigens (Virion\Serion, https://www.virion-serion.de) for phase 1 IgG, phase 2 IgG, and phase 2 IgM run in parallel with previous specimens having negative titers of <1:10. If high titer phase 1 IgG (>1:640) is seen via IFA, then complement fixation for phase 1 and phase 2 total antibodies is performed by using commercial antigens and antisera (Viron\Serion); titers >1:8 are reported as positive.
§Number of days for serologic testing to be repeated after the initial serology.
¶Phase I IgG (by IFA) was persistently ≥1:1,280, but no symptoms of chronic Q fever were observed on serial clinic review, a negative total body positron emission tomography/computed tomography scan, and serial echocardiograms.
#Phase I IgG titer was <1:10 by IFA.
**Diagnostic PCR was performed in the community 1 day before patient attended the hospital.

Appendix Table 3. Selected laboratory findings for the 9 patients requiring ICU admission for acute Q fever in tropical Australia, 2015–2023*

	Patient no.								
Laboratory test	1	2	3	4	5	6	7	8	9
Initial hemoglobin, g/L	154	115	134	139	128	157	143	114	163
Lowest hemoglobin, g/L	127	87	63	134	99	106	86	109	125
Initial leukocyte count, $\times 10^9$/L	6.9	10.4	13.2	7.0	5.2	7.3	11.7	3.9	6.1
Highest leukocyte count, $\times 10^9$/L	11.2	10.4	13.2	10.2	8.0	14.9	11.7	12.3	12.1
Initial neutrophil count, $\times 10^9$/L	5.1	7.7	12.8	6.0	4.8	74.8	9.9	3.8	5.0
Highest neutrophil count, $\times 10^9$/L	7.4	7.7	12.8	7.8	4.8	11.8	9.9	4.4	5.3
Initial platelet count, $\times 10^9$/L	175	58	20	16	79	125	134	71	93
Lowest platelet count, $\times 10^9$/L	172	58	11	16	39	95	134	63	54
Initial serum sodium, mmol/L	138	132	128	135	128	130	136	126	125
Lowest serum sodium, mmol/L	137	127	128	134	126	126	134	126	125
Initial potassium, mmol/L	3.9	3.7	2.5	4.7	3.2	4.3	3.8	2.8	4.0
Lowest serum potassium, mmol/L	3.3	3.7	2.3	4.4	2.9	4.3	3.2	2.8	3.9
Initial serum urea, mmol/L	5.2	6.4	24.0	5.9	4.7	8.4	3.5	5.5	4.3
Highest serum urea, mmol/L	4.7	6.4	24.0	6.7	4.7	14.3	6.9	5.5	1.9
Initial serum creatinine, μmol/L	53	51	86	37	70	84	60	123	88
Highest serum creatinine, μmol/L	59	58	86	42	70	86	60	123	88
Initial serum total bilirubin, μmol/L	14	110	58	95	12	21	10	20	17
Highest serum total bilirubin, μmol/L	14	110	58	173	45	156	13	34	42
Initial serum albumin, g/L	32	22	18	18	19	27	27	24	29
Lowest serum albumin, g/L	25	19	16	18	17	11	19	21	20
Initial serum alanine aminotransferase, IU/L	55	129	29	239	77	125	68	119	151
Highest serum alanine aminotransferase, IU/L	129	129	38	270	118	125	68	243	151
Initial serum aspartate aminotransferase, IU/L	82	272	68	361	107	164	68	234	194
Highest serum aspartate aminotransferase, IU/L	170	272	108	423	196	180	68	272	211
Initial serum lactate dehydrogenase, IU/L	562	624	331	625	668	341	325	557	609
Highest serum lactate dehydrogenase, IU/L	562	624	501	722	886	341	357	557	609
Highest international normalized ratio	1.3	1.2	2.0	1.1	1.7	2.1	1.1	1.4	1.1
Highest C-reactive protein, mg/L	118	227	56	40	190	167	50	197	158
Highest troponin I†	<0.01 μg/L	9.9 μg/L	NT	13 ng/L	5 ng/L	NT	43 ng/L	29 ng/L	NT

*Laboratory values when patient was first seen and the most deranged values during hospitalization are shown. NT, not tested; IU, international units.

†The Beckman Coulter assay for troponin I (reference range <0.040 μg/L) was replaced by the Siemens Atellica assay (reference range <20 ng/L for men and <10 ng/L for women) during the study period.

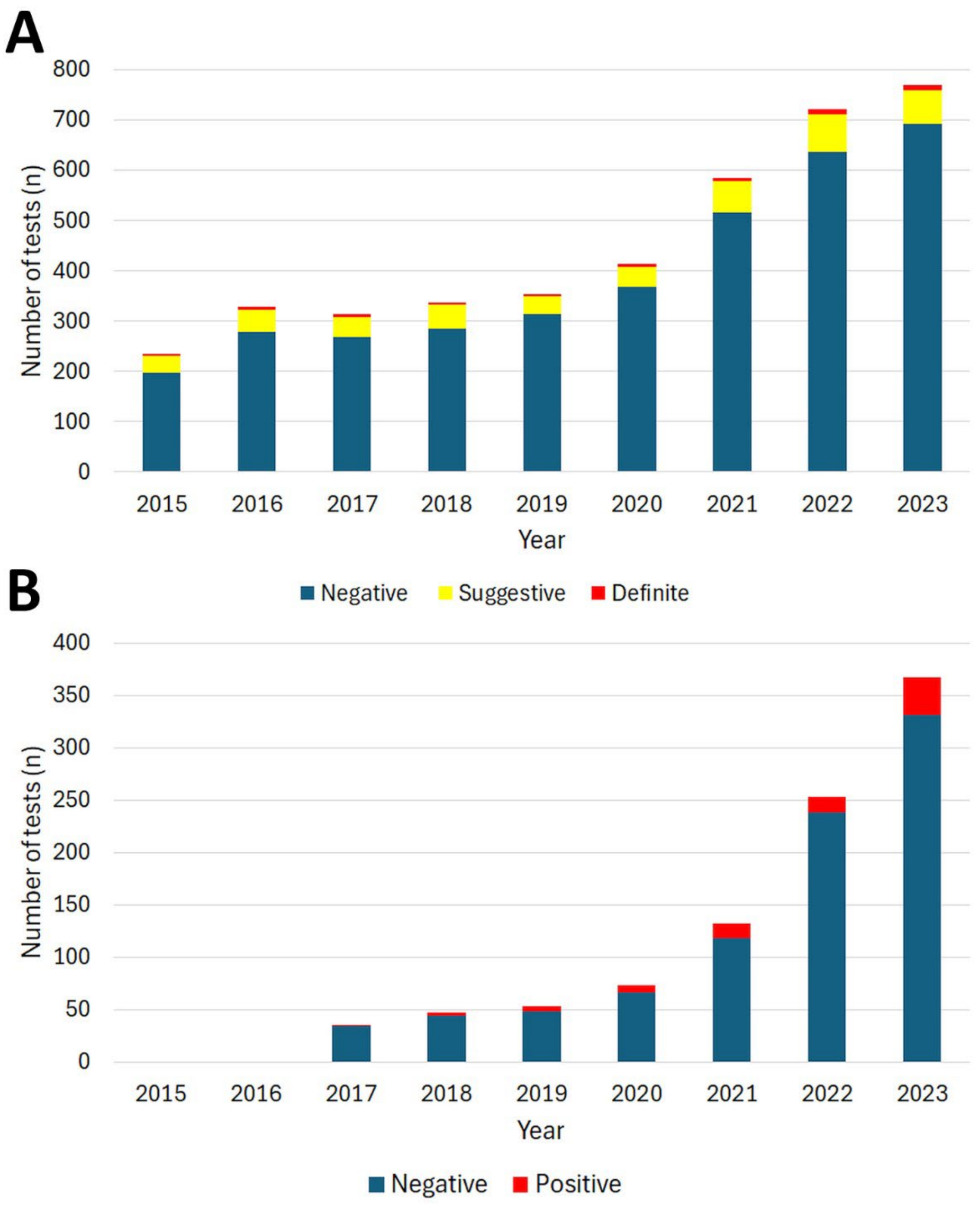

Appendix Figure 1. Testing for Q fever during 2015–2023 in Far North Queensland, tropical Australia. A) Serologic testing of patient samples. B) PCR of patient samples during the study period. Bar colors indicate the results of those tests, defined using the Australia Public Health Laboratory Network laboratory criteria (https://www.health.gov.au/resources/publications/q-fever-laboratory-case-definition).

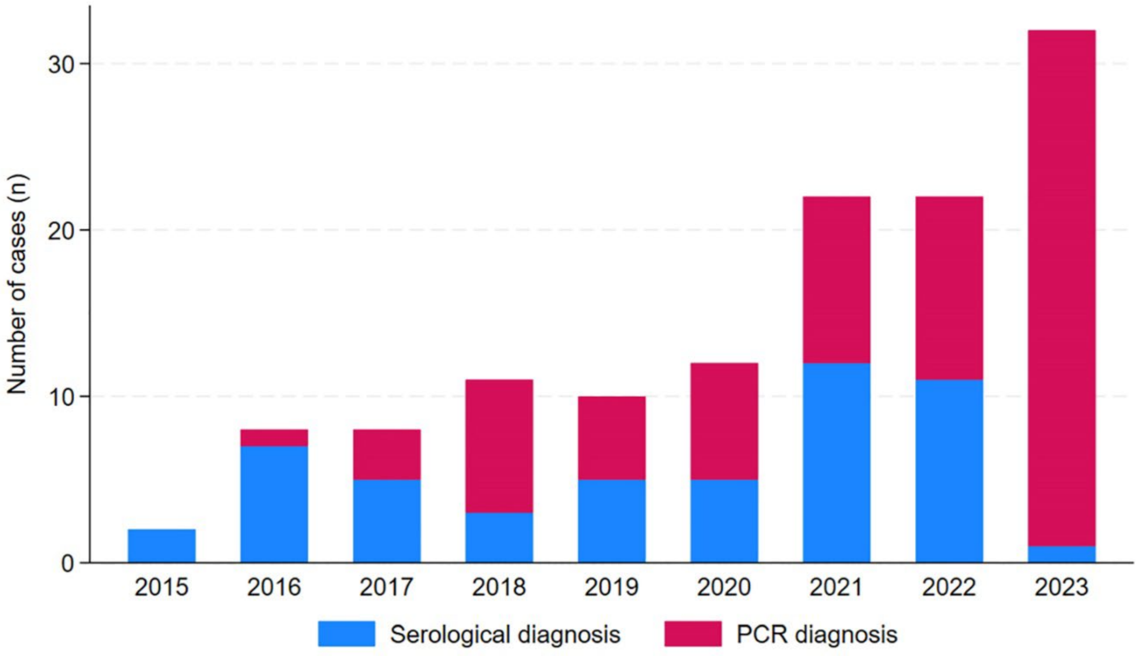

Appendix Figure 2. Method used to diagnose acute Q fever in patients during 2015–2023 in Far North Queensland, tropical Australia. Bar colors indicate whether serologic testing or PCR was used to confirm the diagnosis of the acute *Coxiella burnetii* infection.
